# Expression and prognostic value of tumor stem cell markers ALDH1 and CD133 in colorectal carcinoma

**DOI:** 10.3892/ol.2013.1723

**Published:** 2013-12-02

**Authors:** FEI ZHOU, YIN-DONG MU, JUN LIANG, ZHI-XIN LIU, HONG-SHENG CHEN, JI-FEI ZHANG

**Affiliations:** 1 Department of Anorectal Surgery, Qiqihar Traditional Chinese Medicine Hospital, Qiqihar, Heilongjiang 161000, P.R. China; 2 Department of Histology and Embryology, Mudanjiang Medical College, Mudanjiang, Heilongjiang 157000, P.R. China; 3 Treatment Center of Oncology, The Fourth Affiliated Hospital of Harbin Medical University, Harbin, Heilongjiang 150001, P.R. China

**Keywords:** ALDH1, CD133, colorectal carcinoma, stem cell, cancer stem cell, immunohistochemistry

## Abstract

The identification of cancer stem cells (CSCs) has improved the understanding of tumor occurrence and development. According to CSC theory, colorectal carcinoma (CRC) may be derived from these few cells. Thus, markers for CSCs may lead to the identification of CSCs and investigation of the correlation with various clinicopathological features and survival time in human CRC patients. Aldehyde dehydrogenase 1 (ALDH1) and CD133 (also known as Prominin-1 or AC133) were involved in the current study. The aim of the present study was to identify CSCs through markers of CSCs and to explore the value of the CSC markers, ALDH1 and CD133, in human CRC. The correlation between ALDH1 and CD133 protein expression and the various clinicopathological parameters were investigated through immunohistochemistry (IHC). In addition, the Kaplan-Meier method was used to estimate patients’ overall survival. Correlation of the survival differences between ALDH1- or CD133-positive expression and negative controls was analyzed by the log-rank test. Furthermore, the correlation between the expression of ALDH1 and CD133 was assessed by Spearman’s rank correlation. A marked correlation between the differentiation degree and expression of ALDH1 in tumor cells was demonstrated, but not with CD133 expression. In addition, it was demonstrated that low-stage tumors exhibit a higher expression of ALDH1 or CD133 staining compared with high-stage tumors. Meanwhile, CD133 expression was associated with lymph node metastasis-positive cases, but ALDH1 expression was not. Furthermore, compared with negative cases, ALDH1-positive patients exhibited a poor prognosis. However, no significant difference was identified between CD133-positive and -negative cases in terms of survival time. Overall, the results of the present study indicated that ALDH1 and CD133 may serve as useful markers of CSC to predict disease prognosis and clinicopathological characteristics of human CRC.

## Introduction

Colorectal carcinoma (CRC) is one of the most common types of digestive tract malignant tumor in humans. It is the forth and third most common type of cancer in males and females worldwide, respectively (1), and is one of the major causes of cancer-related mortality (2). CRC incidence rates have continued to increase in economically transitioning countries, which may reflect the adoption of western lifestyles and behaviors, including the consumption of high-fat diets, physical inactivity and smoking (3). The cancer stem cell (CSC) theory was first proposed by Hamburger and Salmon (4) who demonstrated that only a small percentage of tumor cells are able to form colonies in soft agar. According to the CSC hypothesis, cancer originates from uncommon cells, stem cells (SCs), which show pluripotency and self-renewal (5). These self-renewing CSCs may constitute only a small fraction of the tumor cells, with the bulk of the tumor composed of differentiated cells that lack self-renewal capacity (6). These CSCs are hypothesized to cause the initiation, progression and recurrence of cancer. Markers of CSCs may be used to identify CSCs and study their role in the cause of tumorigenesis.

Aldehyde dehydrogenase 1 (ALDH1), a detoxifying enzyme responsible for the oxidation of intracellular aldehydes (7), is one of the common markers of CSCs and SCs. Immunohistochemistry (IHC) results have previously demonstrated that ALDH1 expression and enzyme activity were higher in breast, lung or colon cancer, in which ALDH1 expression was limited in the normal tissue, but was significantly increased in malignant tissue (8–10). The aim of the present study was to identify whether ALDH1 may serve as a valuable marker in CRC patients and whether the detection of ALDH1 may be useful for distinguishing between colorectal carcinogenesis and normal colorectal tissues.

CD133 (also known as Prominin-1 or AC133) is a transmembrane glycoprotein of 865 amino acid, with a total molecular weight of 120 kDa, which is expressed on the surface of apical plasma membrane protrusions of embryonic epithelial structures (11). In a number of previous studies, investigators have used monoclonal antibodies against CD133 for the identification and isolation of a putative CSC population from malignant tumors of the brain (12), prostate (13), liver (14), pancreas (15), lung (16) and colon (17–19). Furthermore, CD133 appears to be the most important colon CSC marker, since subpopulations of CD133^+^ colon cancer cells have demonstrated increased tumorigenic potential in transplantation studies *in vivo* and *in vitro*(20). Other previous studies have also shown that overexpression of CD133 is associated with poor prognosis and distant metastasis in primary colon cancer (20,21).

In the current study, immunohistochemical examination of ALDH1 and CD133 expression was performed to identify whether ALDH1 and CD133 expression was present in patients with CRC. In addition, the correlation between the expression of ALDH1 and CD133 was investigated to understand their role in neoplasia and patient prognosis.

## Materials and methods

### Patients and tissue specimens

The tissues of primary CRCs were obtained from the Department of Pathology at the Red Flag Hospital Affiliated to Mudanjiang Medical College (Mudanjiang, China) between January 2005 and January 2007. Prior to surgery, no patients had received any type of therapy, such as radiation or chemotherapy. Of the total 60 cancer specimens, there were 20 well-differentiated, 20 moderately differentiated and 20 poorly differentiated adenocarcinomas. Normal mucosal specimens ≥5 cm distant from the primary CRCs were obtained from patients with CRC. All tissue samples were fixed in formalin, embedded in paraffin and deparaffinized for IHC staining. All protocols were reviewed and approved by the Ethical Committee of Mudanjiang Medical College (Mudanjiang, China) and written informed consent was obtained from all participating patients. All tumor histology and grades were determined by diagnostic evaluation by two pathologists.

### Follow-up

Clinical and pathological records of all patients involved in the study were reviewed periodically. Patients were followed up regularly for five years at the Red Flag Hospital Affiliated to Mudanjiang Medical College. All patients were followed up from January 1, 2005 to mortality or the study closing date (February 1, 2012). The overall survival (OS) of each case was the assessment used for prognostic analyses.

### IHC

IHC was performed as previously described (22,23). Formalin-fixed, paraffin-embedded human CRC tissue blocks were sectioned at 4-μm thickness. All slides were deparaffinized with xylene and rehydrated with alcohol. Antigen retrieval was achieved by pressure-cooking with target retrieval solution (EarthOx, LLC, San Francisco, CA, USA) for 8 min. The sections were rinsed with TBS and blocked in buffer for 30 min in a wet box at 37°C. For ALDH1 and CD133 staining, sections were respectively incubated at 4°C overnight with anti-ALDH1 and -CD133 solution (1:400; EarthOx, LLC). Slides were then incubated with secondary antibody and, following incubation, sections were rinsed three times with TBS-T. Next, DAB solution was used to colorize the specimens, prior to dehydrating, clearing and mounting with neutral gums. Finally, the samples were examined by microscopy (ECL1 PSE 80i; Nikon, Tokyo, Japan).

### Evaluation of labeling

Imaging analysis of the colorectal tumors for ALDH1 and CD133 expression was performed in three to seven randomly selected high-power fields (magnification, ×200) per case. Staining intensity was scored as follows: 0, negative; 1, weak; 2, moderate; and 3, strong. The positively stained area (distribution) was expressed as the percentage of the whole area under evaluation and scored as follows: 0, no staining; 1, 1–20% positive cells; 2, 21–50% positive cells; 3, 51–80% positive cells; and 4, 81–100% positive cells. It was assured that >20% of tumor cells showing ALDH1 or CD133 staining were positive. Immunohistochemical evaluation of ALDH1 or CD133 expression was performed independently by two pathologists blinded to the patients’ clinical and pathological information. Discrepancies between the pathologists were resolved by consensus.

### Statistical analysis

All data were analyzed using SPSS software for Windows, version 19.0 (SPSS Inc., Chicago, IL, USA). The correlation between the immunohistochemical staining of the markers (ALDH1 or CD133) and the clinicopathological parameters was evaluated by the χ^2^ test, and P<0.05 was considered to indicate a statistically significant difference. The correlation between the expression of ALDH1 and CD133 was assessed by Spearman’s rank test, and P<0.001 was considered to indicate a statistically significant difference. The Kaplan-Meier method was used to estimate the OS of patients, and the correlation between survival differences and expression of ALDH1 or CD133 was analyzed by the log-rank test.

## Results

### Patient characteristics

A total of 60 patients were enrolled in the present study, with 50% males (30 out of 60) and 50% females (30 out of 60). The median age of the patients was 51.6 years (range, 32–68 years) and the male to female ratio was 1:1. Of all patients, 25 (41.7%) were ≥60 years old and 35 (58.3%) were <60 years old. The diagnosis of all patients was colorectal adenocarcinoma. Patients were classified with well-, moderately or poorly differentiated tumor cells. Out of the 60 patients, there were 20 (33.3%) with well-differentiated, 20 (33.3%) with moderately differentiated and 20 (33.3%) with poorly differentiated adenocarcinoma. In total, 24 patients (40%) were at Dukes’ stages A and B, while 36 patients (60%) were at Dukes’ stages C and D. In addition, there were eight (13.3%) TNM stage I, 16 (26.7%) TNM stage II, 28 (46.7%) TNM stage III and eight (13.3%) TNM stage IV patients. Lymph node metastases were present in 34 patients (56.7%) and absent in 26 patients (43.3%) (Table I).

### Expression of ALDH1 and CD133 in normal colorectal and CRC tissue

CRC tissue exhibited significantly higher levels of ALDH1 and CD133 protein expression compared with normal colorectal tissue (P<0.05). ALDH1 and CD133 were detected in the cytoplasm of CRC tissue. In the 60 normal controls, ALDH1 reactivity was demonstrated in ~10% of the epithelial cells and the CD133 expression rate was <20% of the total cells. Of the 60 CRC specimens, high levels of staining were identified in the samples (≥20%). Patients exhibiting >20% positive cells were classified as ALDH1- or CD133-positive and the remainder as negative. The results of immunostaining for ALDH1 and CD133 in the normal colorectal and CRC tissues are shown in Table II.

### Correlation between the positive expression of ALDH1 and CD133 and clinicopathological characteristics of CRC

**Figure 1:**
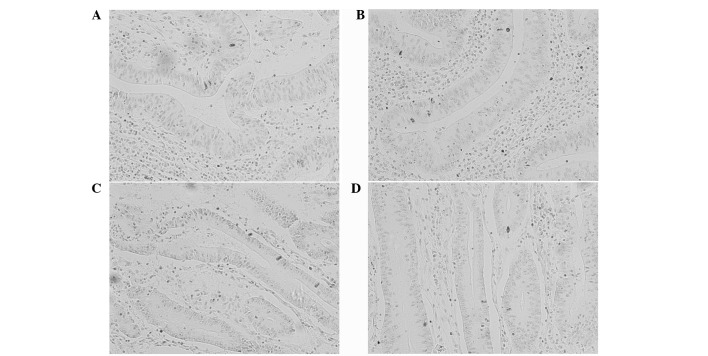
Immunohistochemical staining of ALDH1 in colorectal carcinoma cells. Expression of ALDH1 in (A) well-differentiated, (B) moderately differentiated, (C) TNM stage II and (D) TNM stage III tumor cells. Magnification, ×200. ALDH1, aldehyde dehydrogenase 1.

**Figure 2:**
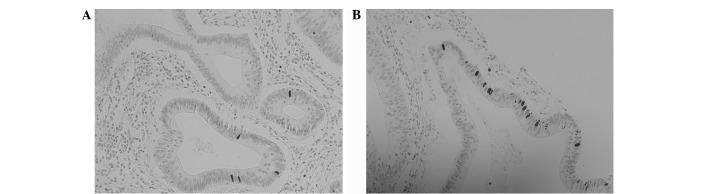
Immunohistochemical staining of CD133 expression in different TNM stages of colorectal carcinoma: TNM stages (A) I and (B) III. Magnification, ×200.

No significant correlation was identified between ALDH1 and CD133 expression and patient age, gender and tumor size. ALDH1 expression was closely associated with tumor cell differentiation and Dukes’ and TNM staging. It was found that with an improved degree of differentiation, the tumor cells exhibited a lower rate of ALDH1 staining. A marked positive correlation was observed between the degree of differentiation and proportion of ALDH1 immunostaining in tumor cells (χ^2^=8.918; P<0.05) among the tested samples. A higher ALDH1 band intensity was detected in poorly differentiated malignant tumor cells compared with the well- or moderately differentiated tumor cells (Figs. 1A and B). ALDH1 expression was noted in 5 (25%) of the 20 well-differentiated cases, 12 (60%) of the 20 moderately differentiated cases and 14 (70%) of the 20 poorly differentiated cases. Patients with Dukes’ stages C and D showed a higher expression rate of ALDH1 (24/36; 66.7%) compared with those with Dukes’ stages A and B (7/24; 29.2%) (P<0.05). Patients with TNM stages III and IV exhibited greater positive staining for ALDH1 compared with those with TNM stages I and II, similar to Dukes’ stages C and D compared with Dukes’ stages A and B (Figs. 1C and D). No association was identified between lymph node metastasis and ALDH1 expression. By contrast, CD133 expression exhibited no association with tumor cell differentiation, but its expression was closely associated with lymph node metastasis. Positive lymph node metastasis was identified in 20 of the 34 CD133-positive cases (58.8%); however, negative lymph node metastasis was identified in only eight of the 26 CD133-positive cases (30.8%; P<0.05). Furthermore, CD133 expression exhibited a correlation with Dukes’ and TNM staging. The expression of CD133 in Dukes’ stage C and D or TNM stage III and IV was evidently higher compared with that in Dukes’ stage A and B or TNM stage I and II adenocarcinomas (χ^2^=4.92; P<0.05; Fig. 2). The correlation between the ALDH1 and CD133 proteins and clinicopathological features of CRC patients are summarized in Table III.

### Correlation between ALDH1 and CD133 expression in human CRC

Of the total 60 human specimens, ALDH1- and CD133-positive expression was identified in 12 cases, while negative expression was identified in 15 cases. In addition, 19 patients were identified as ALDH1-positive, but CD133-negative. By contrast, 14 patients were identified as ALDH1-negative and CD133-positive. Spearman’s rank correlation analysis showed that ALDH1 expression and CD133 expression in CRC are significantly positively correlated (r=0.241; P=0.0322; Table IV).

### Kaplan-Meier survival analysis

**Figure 3:**
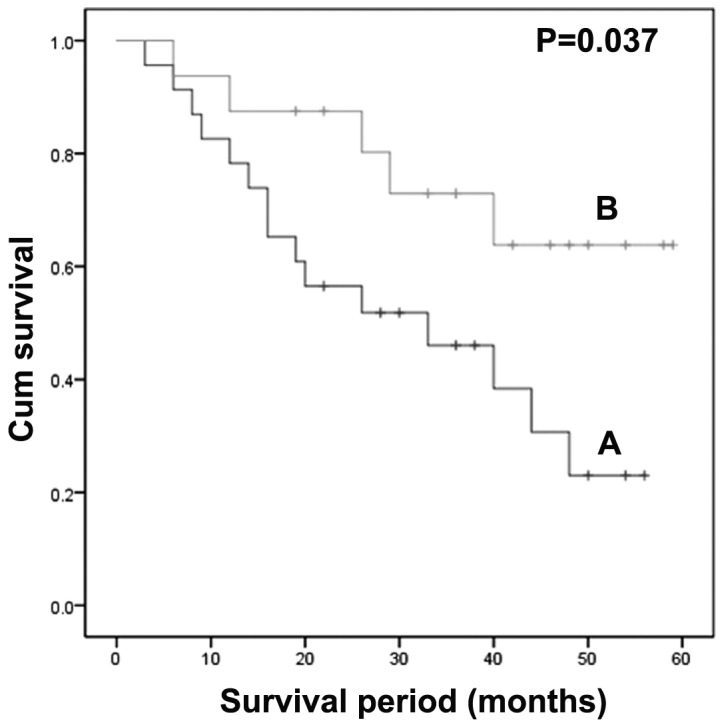
Kaplan-Meier survival curves of colorectal cancer patients with (A) ALDH1-positive (n=31) and (B) ALDH1-negative (n=29) expression. ALDH1, aldehyde dehydrogenase 1.

**Figure 4:**
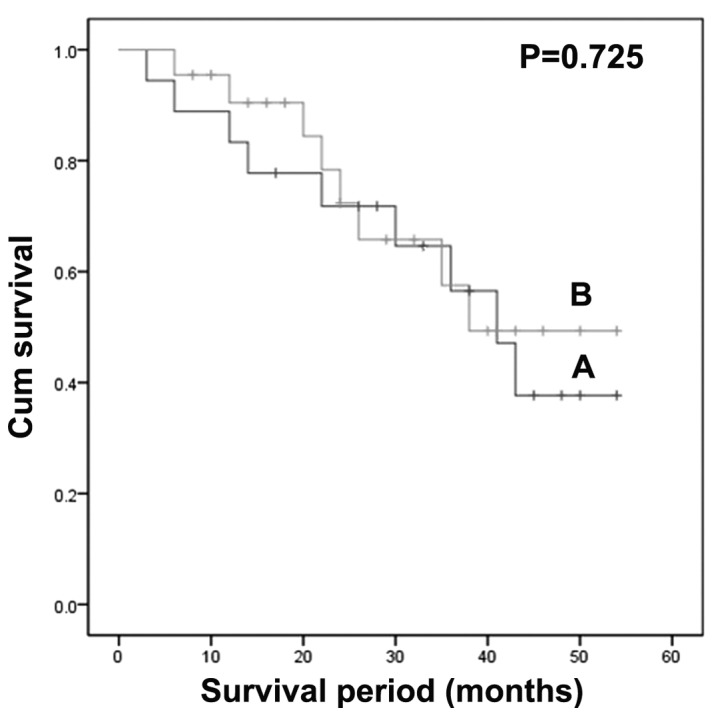
Kaplan-Meier survival curves of colorectal cancer patients with (A) CD133-positive (n=28) and (B) CD133-negative (n=32) expression.

Among the 60 study patients, ALDH1-positive patients showed shorter survival times compared with ALDH1-negative patients (log-rank test; χ^2^=4.34; P=0.037; Fig. 3). However, by contrast, CD133-positive patients did not have significantly higher survival times than CD133-negative patients (log-rank test; χ^2^=0.124; P=0.725; Fig. 4).

## Discussion

CRC is one of the most common types of malignant tumor worldwide (1). Furthermore, it is a complicated and multifactorial process. Hamburger and Salmon first proposed the CSC theory in 1977 (4) and demonstrated that only a small percentage of tumor cells have the capability to form colonies in soft agar. Therefore, therapies are only required to target CSCs. According to the CSC hypothesis, cancer originates from uncommon cells, SCs, which show pluripotency and self-renewal (5). CSCs are hypothesized to cause the initiation, progression and recurrence of cancer. Fractionation of cancer cells on the basis of displayed CSC surface markers has yielded subpopulations of neoplastic cells with a greatly enhanced ability, relative to the majority of corresponding populations, to seed new tumors upon implantation in immunodeficient mice.

ALDH1 is a common expression marker of CSCs and SCs (8). The present study found that compared with normal colorectal tissues, tumor tissues expressed a high ALDH1 staining rate (≥20%; P<0.05). Additionally, a marked correlation between the differentiation degree and expression of ALDH1 was demonstrated. Through IHC staining, it was demonstrated that as the differentiation degree worsened (from well- to poorly differentiated), the ALDH1 staining rate increased (χ^2^=8.918; P<0.05). These results are consistent with a previous study by Huang *et al*, who confirmed that the overall proliferative cell population (SCs and rapidly proliferating cells) increases during colorectal tumorigenesis (8). An additional important observation of the present study was that low-grade tumors exhibited a higher expression of ALDH1 staining compared with high-grade tumors (P<0.05). This result was consistent with Dukes’ staging in the current study. It was also demonstrated that the ALDH1-positive patients exhibited shorter survival times than ALDH1-negative patients (P=0.034). These results imply that ALDH1 may serve as a marker for CSCs to distinguish colorectal carcinogenesis from normal colorectal tissues and to anticipate patient prognosis.

In the present study, CD133 was found to be infrequently expressed in normal colorectal tissues compared with the tumor tissues. This result is consistent with that of a previous study by O’Brien *et al*(17). In addition, the present study identified that age, gender, tumor size and histological grade were independent of CD133 expression levels, consistent with a previous study by Horst *et al*(24). However, it was found that CD133 closely correlates with tumor stage or Dukes’ stage; the higher the stage, the higher the rate of CD133 staining. Specimens positive for lymph node metastasis demonstrated higher expression rates of CD133 compared with negative cases. Although, through survival analysis, no significant correlation was found between the expression of CD133 and patient survival period. This result contradicts that of a previous study by Ieta *et al*(20) demonstrating that overexpression of CD133 was correlated with a poor prognosis. However, the results of the present study show that CD133-negative expression also affected patient survival times, which is consistent with Schmelkov *et al*, who previously demonstrated that CD133-negative colon cancer cells also have the ability to initiate tumors (25).

In conclusion, ALDH1 and CD133 as markers of CSCs are found in CRC tissues. Their expression is closely associated with the prognosis and clinicopathological characteristics of patients with CRC. However, whether the use of a single CSC marker to identify CSCs is sufficient, remains an important question. Therefore, the joint detection of CSC marker expression in patients is likely to be useful to improve predictions for the prognosis of the disease and understanding of the clinicopathological characteristics. Future studies are required to conduct further studies on larger samples, since few CSCs cause tumor relapse and metastasis. The specific markers of CSCs not only accurately detect ‘real’ CSCs, but also develop a novel treatment strategy targeting CSCs in human CRC.

## Figures and Tables

**Table I:** Patient characteristics.

Clinical data	Value
Patients, n (%)	60 (100.0)
Male	30 (50.0)
Female	30 (50.0)
Age, years	
≥60, n (%)	25 (41.7)
<60, n (%)	35 (58.3)
Median (range)	51.6 (32–68)
Differentiation, n (%)	
Well	20 (33.3)
Moderate	20 (33.3)
Poor	20 (33.3
Dukes’ stage, n (%)	
A and B	24 (40.0)
C and D	36 (60.0)
TNM stage, n (%)	
I	8 (13.3)
II	16 (26.7)
III	28 (46.7)
IV	8 (13.3)
Lymph node metastasis, n (%)	
Positive	34 (56.7)
Negative	26 (43.3)

**Table II:** Comparison of ALDH1- and CD133-positive expression between colorectal cancer and normal colorectal tissues.

Colorectal tissue	n	ALDH1-positive, n (%)	χ^2^	P-value	CD133-positive, n (%)	χ^2^	P-value
Cancer	60	31 (51.7)			28 (46.7)		
Normal	60	15 (25.0)	4.51	<0.05	13 (21.7)	4.168	<0.05

ALDH1, aldehyde dehydrogenase 1.

**Table III:** Correlation between the positive expression of ALDH1 and CD133 and clinicopathological characteristics of human colorectal cancer.

Variables	n	ALDH1-positive, n	χ^2^	P-value	CD133-positive, n	χ^2^	P-value
Gender							
Male	30	17	0.6000	>0.05	15	0.2678	>0.05
Female	30	14			13		
Age, years							
≥60	25	15			13		
<60	35	16	1.1910	>0.05	15	0.4898	>0.05
Tumor size, cm							
≤3	22	10			13		
>3	38	21	0.5370	>0.05	15	2.1500	>0.05
Differentiation							
Well	20	5			12		
Moderate	20	12	8.9180	<0.05	10	3.7500	>0.05
Poor	20	14			6		
Dukes’ stage							
A and B	24	7			7		
C and D	36	24	8.1000	<0.05	21	4.9200	<0.05
TNM stage							
I and II	24	7			7		
III and IV	36	24	8.1000	<0.05	21	4.9200	<0.05
Lymph node metastasis							
Positive	34	17			20		
Negative	26	14	0.0873	>0.05	8	4.659	<0.05

ALDH1, aldehyde dehydrogenase 1.

**Table IV:** Correlation between ALDH1 and CD133 expression and colorectal cancer.

	CD133			
			
ALDH1	Positive	Negative	r value	P-value
Positive	12	19		
Negative	14	15	0.241	0.0322
Total	26	34		

ALDH1, aldehyde dehydrogenase 1.
